# Investıgatıon of seam performance and bıodegradabılıty of organıc cotton clothes for theır sustaınabılıty

**DOI:** 10.1007/s10532-025-10112-w

**Published:** 2025-02-05

**Authors:** Hatice Kübra Özbey, Ayça Gürarda

**Affiliations:** 1 Vocational School of Technical Sciences, FashionDesign Program, Bursa Uludag University, Görükle, Bursa, Turkey; 2 Faculty of Engineering, TextileEngineeringDepartment, Bursa Uludag University, Görükle, Bursa, Turkey

**Keywords:** Cotton, Organic, Cloth, Biodegradability, Seam performance, Sustainability

## Abstract

In recent years, it has become important for a sustainable environment that clothes are biodegradable, and that unused clothing does not pollute the environment by decomposing in nature. Cotton, especially organic cotton, can decompose very quickly in nature. Since organic clothing production has become important in recent years, the seam performance and biodegradability of these products, unlike conventional products, are important in creating new data for the textile and clothing industry. This paper presents an experimental study of the seam performance and biodegradability properties of organic clothes for their sustainability. In this study, six groups of fabrics with a plain weave structure were produced to examine the seam performance and biodegradability of clothes made of organic cotton woven fabrics. Each group includes three different samples of the fabric: conventional cotton fabric dyed with reactive dyes, organic cotton fabric dyed with reactive dyes, and organic cotton fabric dyed with natural dye. Therefore 18 samples having different properties were obtained. Fabric breaking strength, seam strength, seam efficiency and biodegradability tests of these fabric samples were determined. Sample women's blouses were sewn from the fabric samples used in the experimental study, and the blouse appearances were visually presented on the mannequin.

## Introduction

Organic cotton has become increasingly popular in recent years as consumers prioritize sustainability in their fashion choices. Using sustainable materials plays a vital role in every stage of organic clothing production. Therefore, in organic clothing manufacturing, production steps that comply with the standards must be applied from fiber production to the clothes being offered to the market (Delate et al. 2021). Since organic clothing manufacturing is a new field, unlike conventional products, performance tests to be conducted on these products are essential in creating new information for the textile and clothing sector. Organic cotton is grown using 71% less water and 62% less energy than conventional cotton. Buying organic cotton clothes protects water, air, soil, and the health of farmers and employees. Sustainable fashion has a production chain that saves energy and water. It respects the environment and employees, uses renewable energy, and produces clothes free of hazardous chemicals. Sustainable fashion uses natural, organic, and recycled fabrics.

Organic clothing production is very important for sustainability in the textile and clothing sector. In order for organic clothing production to become widespread, the seam performance and biodegradability of these clothes must be analyzed very well and compared with conventionally produced clothes. Seam efficiency is very important in determining seam performance. Seam efficiency is defined as the capacity of the material itself to carry a seam. Seam efficiency can be optimised through different factors, such as fabric structure, seam type, type and density of stitches and the selection of sewing threads and needles. Seam efficiency values between 60 and 80% are typical for clothes. Low seam efficiency values indicate the sewn fabric is damaged during sewing (Laing and Webster 1998; Gurarda 2014).

In recent years, in order to prevent environmental pollution, clothes have begun to be produced with biodegradability in mind. This means using nature-based, nature-friendly and easily biodegradable materials. Organic cotton is made of 100% natural cellulose fibers, making it biodegradable and compostable. The thread count of organic cotton fabric can vary widely, depending on the specific product and weave. Higher thread counts generally result in a softer, smoother fabric, while lower thread counts may be more breathable and lightweight. Organic textiles are obtained by processing organically produced fibres according to specific standards, starting from the first stage, fiber production. Today, clothing companies are interested in sustainable and ecological fashion. Various standards are developed, and sustainable materials are used in production lines (Ali and Sarwar 2010). Sustainability is achieved in textiles with the concept of textile ecology. Textile ecology is examined in three groups: production, human ecology, and waste ecology. Accordingly, products should be made according to ecological ethics, should not harm human health, and clothing or organic production is considered environmental (Can and Ayvaz 2017).

More than 8,000 chemicals are used in producing clothes, and these return to nature as chemical waste. Since there is no use of GMO (genetically modified organism) raw materials, chemicals, pesticides, or radiation in the production of organic products, no factors that will negatively affect human health and nature afterwards (Kalkancı 2017; Akbulut 2012).

Among the standards of organic products, GOTS and OCS (Organic Content Standard)/OE (Organic Exchange) certificates stand out in the sector. The GOTS standard prioritises the environment and people in every area, from the production stages of the fiber to the shipment of the final product, as well as social rights in working conditions. Buying GOTS-certified textiles and clothes shows that you have a sustainable social responsibility that causes minimum harm to the environment. GOTS-certified products have different label grades. Products bearing the "Organic" label grade contain a minimum of 95% certified organic fibres. These fibres are sourced from organic farming practices, meeting the highest standards of environmental and social responsibility. The "Made with Organic materials" label grade indicates that at least 70%, up to 95% of the product's fibres are certified organic. This grade allows for a balance between organic and conventional fibres while maintaining a strong commitment to sustainability (Global Organic Textile Standards 2023).

The OCS standard examines whether the product is organic in production stages, such as fiber and chemicals, throughout the supply chain. The OCS applies to products that contain at least 5% organically grown material, calculated as a percentage of the entire product, excluding accessories and trims (Organic Content Standard 3.0 2020).

The Organic Exchange Blended standard monitors and documents the purchase, processing, and use of certified organically grown fiber in yarns, fabrics, and finished products. Non-organic cotton, synthetic, and regenerated fibers can be used in the remaining blend. To have the Organic Exchange 100 certificate, the company must use at least 95% organic cotton fibre in its product. Non-organic cotton is not used in these products, and regenerated and synthetic fibers can be used in the remaining 5%. If the product contains at least 95% organic cotton, the label should state ‘‘made from organically grown cotton.’’ If 100% organic cotton is used, the label should state "made from 100% organic cotton” (Altun 2012).

High amounts of water, energy, chemical and other related sources are consumed at the stage of processing cotton fibers into textile products. Especially dyeing process utilized in textiles may lead to environmental pollution due to its chemical dyestuff. As a remarkable approach to sustainable textiles, the usage of naturally coloured organic cotton fiber and converting it into textile materials are one of the promising methods which provide a reduction of the environmental hazard (Gunaydın et al. 2020).

Dyeing of organic cotton is an important process step in textile production. Mainly, reactive, direct or vat dyes are used. Natural dyes are offered as a more sustainable alternative to synthetic dyes in the textile industry. Dye pigments obtained from plants, insects, animals, or minerals are known as natural dyes (Kalaivaanee and Abirami 2014). The issues surrounding synthetic dyes are well-known, and in the literature, it is generally accepted that natural dyes have a smaller environmental impact. However, even natural dyes have issues that must be dealt with in order to ensure sustainability in the industry. For example, the mordants needed to improve the dyeing process may release hazardous heavy metal pollutants when used (Muthu 2023; Kadınkız et al. 2023). Sustainable use of natural dye in the textile industry is achieved by connecting economic, social and environmental aspects by increasing creativity in technology, increasing capacity and productivity, and ensuring supply chain sustainability ( Elsahida et al. 2019).

Natural dyes might be promising alternatives to synthetic dyes for non-alergic, non-toxic and eco-friendly characteristics. So, the uses of natural dyes are potentially viable “Green Chemistry” for preventing the hazardous synthetic dyes for their various growing environmental and health concerns (Repon et al. 2018). For the successful use of natural dyes, appropriate and standardized dyeing techniques must be applied (Samantaa and Agarwal 2009). The natural dyes produce different colours and shades. Compared to cotton, organic cotton dyed with natural dyes is safe and eco-friendly. Therefore, their use will definitely minimize the health hazards caused by the use of synthetic dyes. A comparison of the properties of the cotton and organic cotton samples, such as durability, strength and absorbency, shows that the organic cotton sample is much better than the cotton sample (Ramasamy 2015).

As a result of these explanations, six groups of fabric samples were examined in this study. Each group includes reactive-dyed conventional fabrics, reactive-dyed organic cotton fabrics and naturally dyed organic fabrics. The fabric breaking strength, seam strength, seam efficiency and biodegradability of these samples were tested. Sample women's blouses were sewn from the fabric samples used in the experimental study, and the blouse appearances were visually presented on the mannequin. Another aim of this study is to compare the performance properties and biodegradability of organic cotton woven fabrics with those produced conventionally and to reveal the advantages and disadvantages of clothes made of organic cotton woven fabrics. It is thought that when the advantages and disadvantages of organic clothing are revealed, consumer demand for these clothes will also increase.

## Material and method

Six groups of fabrics with a plain weave structure were produced to examine the performance properties and biodegradability of clothes made of organic cotton woven fabrics. Each group includes three different samples of the fabric: conventional cotton fabric dyed with reactive dyes, organic cotton fabric dyed with reactive dyes, and organic cotton fabric dyed with thuja oak natural dye. In natural dyeing, pre-mordanting was first done in a hot bath. Then dyeing was done in a hot bath at 60 °C. Warm and cold rinsing was done. All chemicals used during dyeing are natural. Thus, 18 fabric samples with different properties were obtained. Table 1 shows the structural properties of the fabric samples.

**Table 1:** Structural properties of fabric samples

Fabric code	Organic certificate /dye type	Average weight (g/m^2^)	Yarn count (Ne), *(den)		Composition (%)
			Weft	Warp	
1a	Uncertified-Reactive				
1b	Organic GOTS-Reactive	137	40/1	40/1	%100 CO
1c	Organic GOTS-Natural				
2a	Uncertified-Reactive				
2b	Organic GOTS-Reactive	94	80/1	80/1	%100 CO
2c	Organic GOTS-Natural				
3a	Uncertified-Reactive				
3b	Organic OCS-Reactive	65	50*CV	80/1	%54 CO + %46* CV
3c	Organic OCS-Natural				
4a	Uncertified-Reactive				
4b	Organic GOTS-Reactive	155	40/1	62/1	%100 CO
4c	Organic GOTS-Natural				
5a	Uncertified-Reactive				
5b	Organic GOTS-Reactive	191	80/2	80/2	%100 CO
5c	Organic GOTS-Natural				
6a	Uncertified-Reactive				
6b	Organic OCS-Reactive	116	40/1	40*PA + 20*EL	%67CO + %27*PA + %4* EL
6c	Organic OCS-Natural				

Fabric breaking strength (TS EN ISO 13934–1, 2013), seam strength, seam efficiency (ASTM 1683–81, 2017) and biodegradability tests were performed on these fabric samples.

Seam strength can be measured according to ASTM D 1683–81, "Standard Test Method for Failure in Sewn Seams of Woven Apparel Fabrics". This test method measures the sewn seams’ strength in woven fabrics by applying a force perpendicular to the sewn seams (ASTM 1683–81, 2017).

Conventional samples were sewn with Tkt 60 polyester core spun sewing thread; organic samples were sewn with Tkt 60 Coats Tre Cerchi Vero cotton thread, which is ecological with the Better Cotton certificate, with 301 SSa-1 lockstitch and five stitch/cm stitch density.

Seam efficiency is defined as the material's ability to carry seams. Seam efficiency is the ratio of seam strength to fabric strength. Equation 1 was used to calculate the seam efficiency.

1 $$Seam \, efficiency \, \left( \% \right) \, = \, 100 \, x \, \left( {seamed \, fabric \, strength \, / \, Unseamed \, fabric \, strength} \right)$$

The biodegradability test was applied to the cotton fabric samples according to the ISO 11721–1:2001 and ISO 11721:2003 soil burial test standards. The biodegradation of fabrics was done by burying the samples in the soil at different times. According to ISO 11721, the samples were cut into 5 × 5 cm dimensions and buried in the soil in 1000 ml beakers. The water content of the test soil is 60 ± 5% of its maximum moisture-holding capacity. Afterwards, the beakers containing the embedded samples were kept waiting for 1,2,3 and 4 weeks. Incubation of soil burial samples was carried out at 29 °C and 95–100% relative humidity.

After the specified burial time, the samples were removed from the soil, washed in ethanol/water (70% / 30% volume fraction) solution for approximately 10 min, and dried at room temperature (ISO 11721–1, 2001; ISO 11721, 2003; Arshad and Mujahid 2011).

Sample women's blouses were sewn from the fabric samples used in the experimental study, and the blouse appearances were visually presented on the mannequin. The same pattern and size 36 were used in all blouses sewn.

SPSS 14.0 statistical program was used to evaluate the test results. Randomized two-factor analysis of variance (two-way-ANOVA) was also performed to determine the statistical significance of fabric weight and fabric organic certificate/ dye type on some mechanical properties such as breaking strength, overall flexural rigidity, seam strength and seam efficiency properties of the fabric samples. The means were compared by means of SNK tests. The value of the significance level (α) selected for all statistical tests in the study was 0.05. The treatment levels were marked in accordance with the mean values, and levels marked by a different letter (a, b, c, d, e) indicate that they were significantly different.

## Results

### Fabric breaking strength test results of fabric samples

**Fig. 1:**
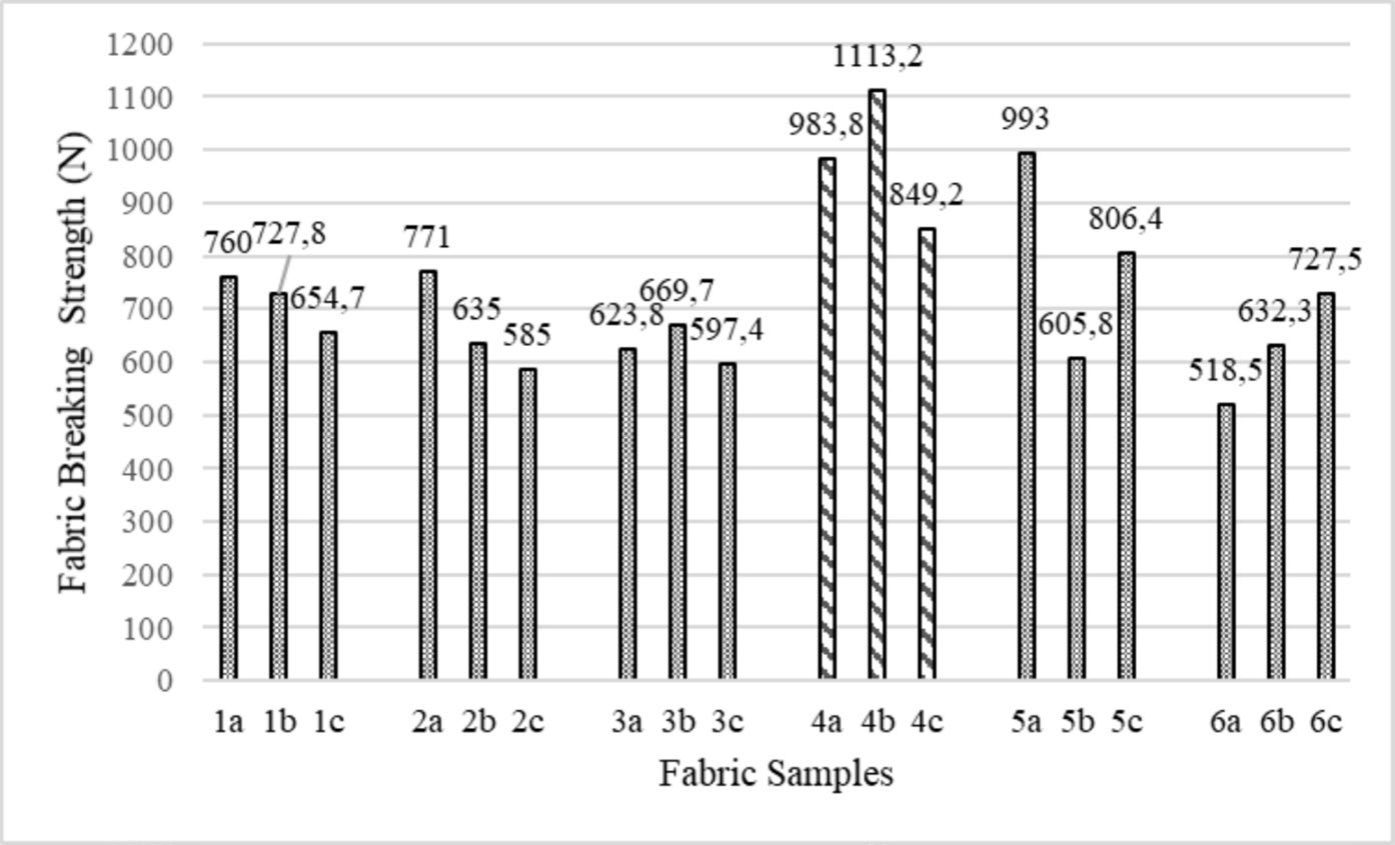
Breaking strength results of the fabric samples

Figure 1 shows the breaking strength results of the fabric samples. As can be seen from the results obtained, the breaking strength of the organic fabric + natural dyed fabric samples is lower. The breaking strength of the natural dyed samples is lower because they do not contain any chemical substances. It is expected that reactive dyeing in an alkaline environment will affect the strength values of cotton fabrics less than natural dyeing in an acidic environment (Elibüyük et al. 2019). Caustic soda is widely used in the reactive dyeing processes. Soda increases pH and level activates the molecules of the fabric so that they can better absorb the dye. This results in brighter and longer-lasting colors. Caustic soda is also used in the mercerizing process to increase the strength, luster, and dye affinity of the fabric. Since fabric groups four and five have the highest weight, their breaking strengths are also high. Fabric sample group three has the lowest weight, and its breaking strength is also low.

The Anova and SNK test results in Table 2 revealed that the fabric samples' weight and organic certificate/dye type are statistically significant on the fabric breaking strength. Fabric groups with high weight (4th and 5th groups) have higher fabric breaking strength than fabric groups with low weight. Fabric samples with organic certificate/reactive dye and organic certificate/natural dye have lower breaking strength than uncertificated/reactive dye fabric samples.

**Table 2:** Statistical analysis (ANOVA and SNK test) results for fabric breaking strength

		Fabric breaking strength	
		P/Sig	SNK Ranges
Weight (g/m^2^)	1st group	.000*	714.22 b
	2nd group		663.80 b
	3rd group		630.29 a
	4th group		982.06 d
	5th group		801.74 c
	6th group		626.13 a
Organic certificate/ dye type	Uncertified-Reactive	.000*	775.10 b
	Organic GOTS/OCS-Reactive		730.62 a
	Organic GOTS/OCS- Natural		703.39 a

^*^: Statistically significant (P < 0.05)

(a), (b), (c) and (d) represent statistically difference ranges according to the SNK test

### Seam strength test results of conventional fabric samples

**Fig. 2:**
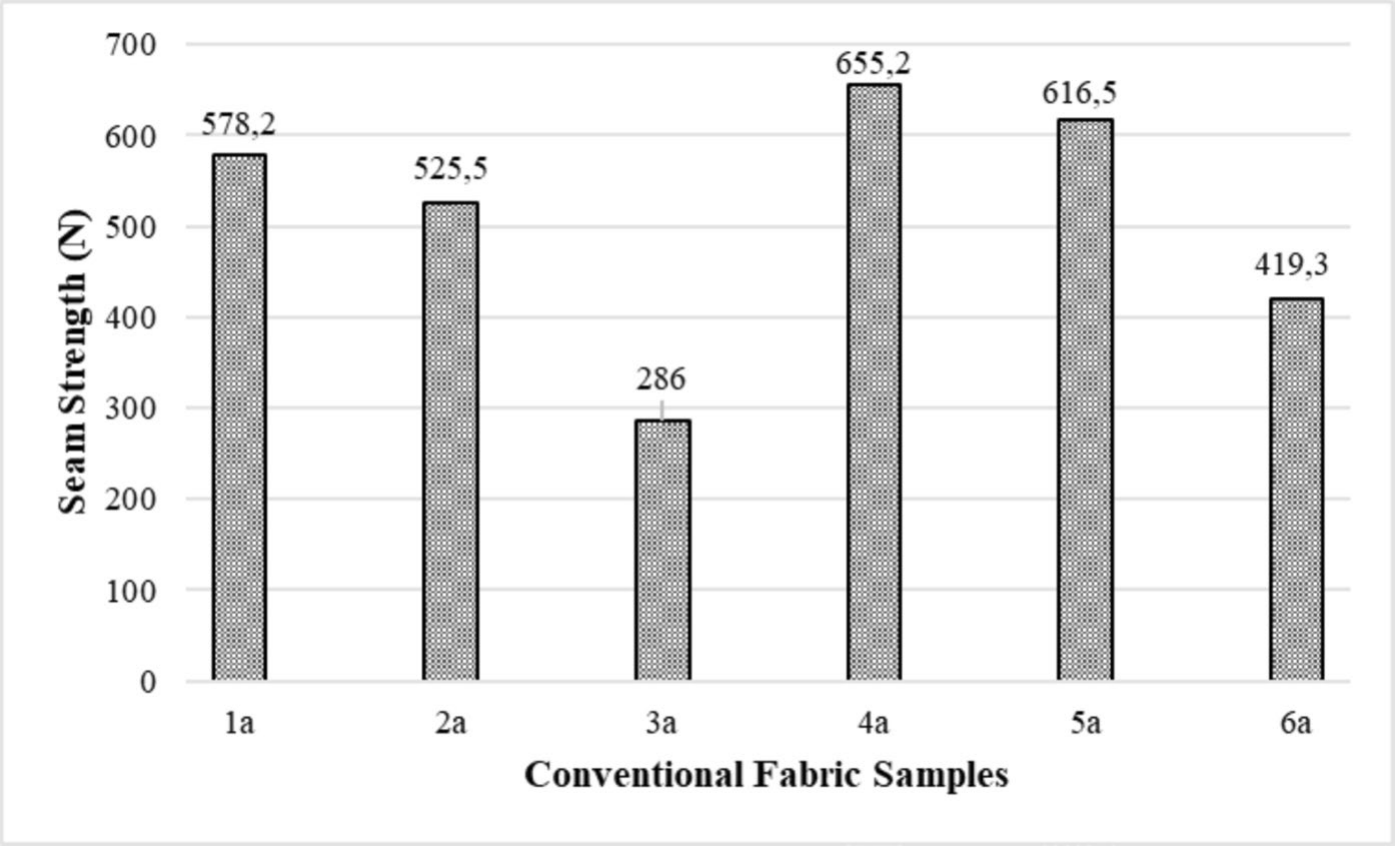
Seam strength results of the conventional fabric + reactive dyed samples

The seam strength results of the conventional fabric + reactive dyed samples used in the experimental study are given in Fig. 2. Since fabric samples 4a and 5a are the highest weight, their seam strengths are also high. Fabric sample 3a has the lowest weight, and its seam strength is also low.

### Seam efficiency test results of conventional fabric samples

**Fig. 3:**
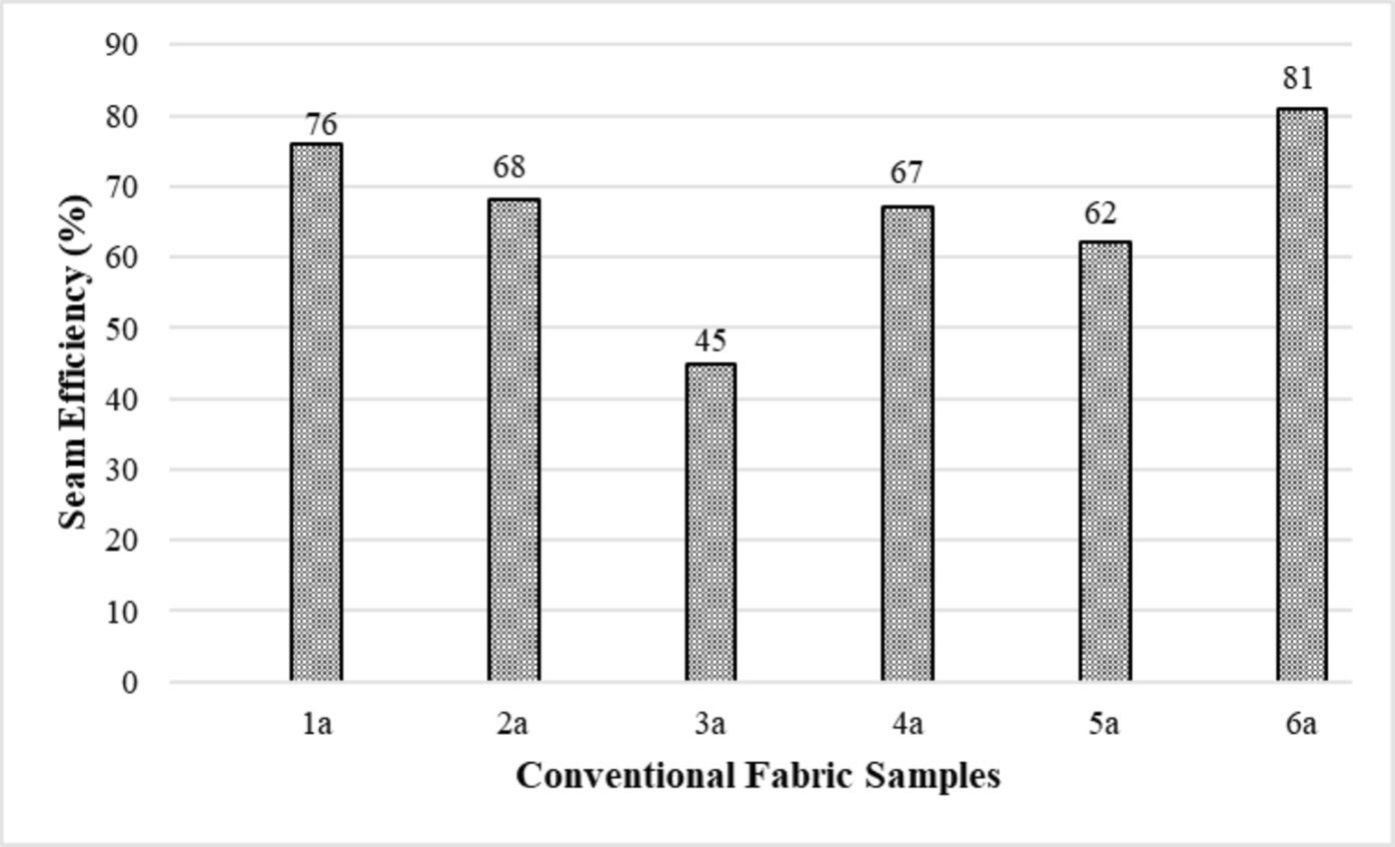
Seam efficiency results of the conventional fabric + reactive dyed samples

Figure 3 shows the seam efficiency values of conventional fabric + reactive dyed samples. The seam efficiency values of conventional fabric + reactive dyed samples were between 45 and 81%. The seam efficiency value of sample 3a, which has the lowest fabric breaking strength and seam strength, was also low, so its sewability was low compared to other samples. The seam efficiency of sample 6a was found to be high.

### Seam strength test results of organic fabric samples

**Fig. 4:**
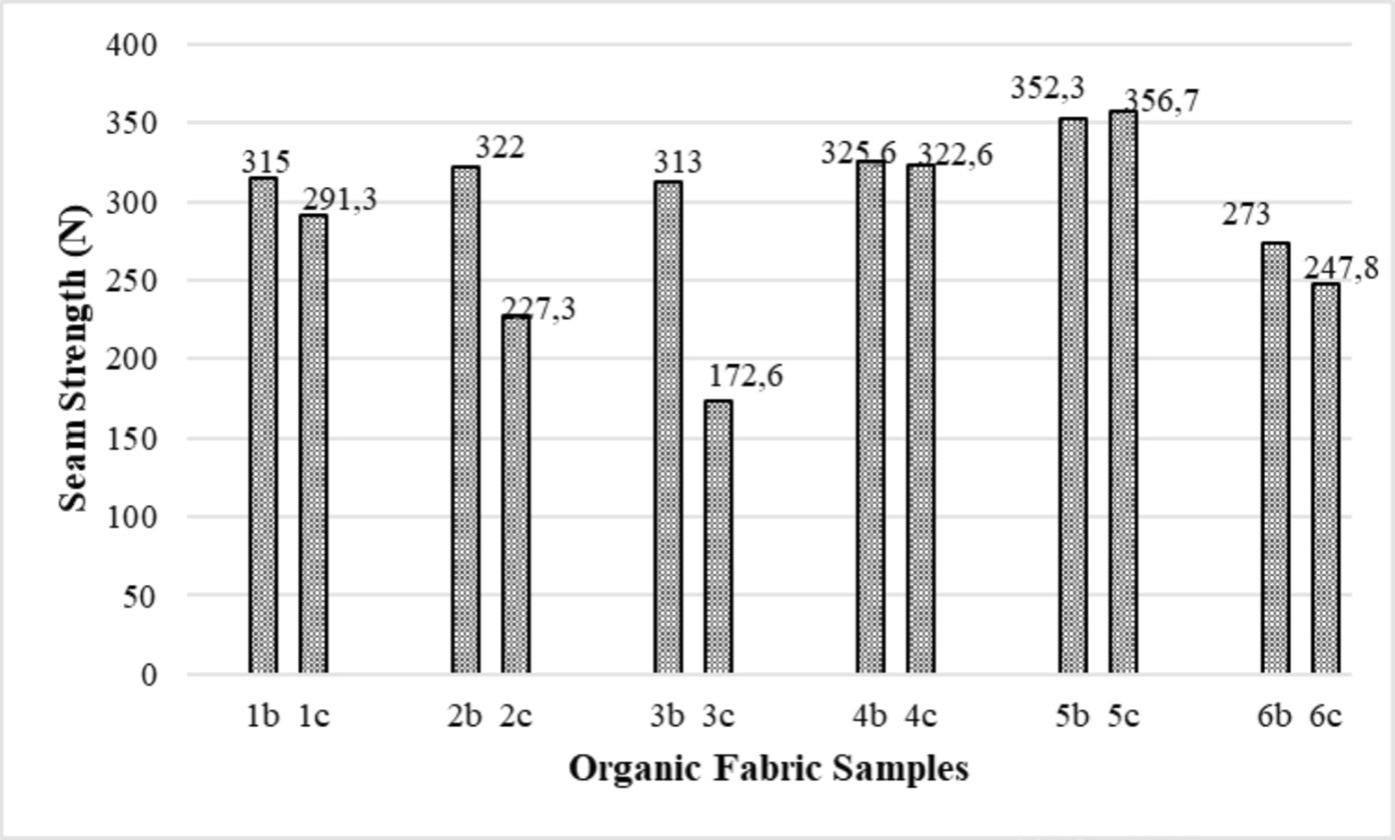
Seam strength results of the organic fabric samples

The seam strength results of the organic fabric + reactive dyed and organic fabric + natural dye samples used in the experimental study are given in Fig. 4. Since fabric samples 4b, 4c and 5b, 5c are the highest weight, their seam strengths are also high. Fabric samples 3b, 3c and 6b, 6c have the lowest weight, and their seam strengths are also low. Cotton sewing thread is used in organic cotton clothes. For this reason, it has lower seam strength than polyester sewing thread used in conventional cotton clothes.

### Seam efficiency test results of organic fabric samples

**Fig. 5:**
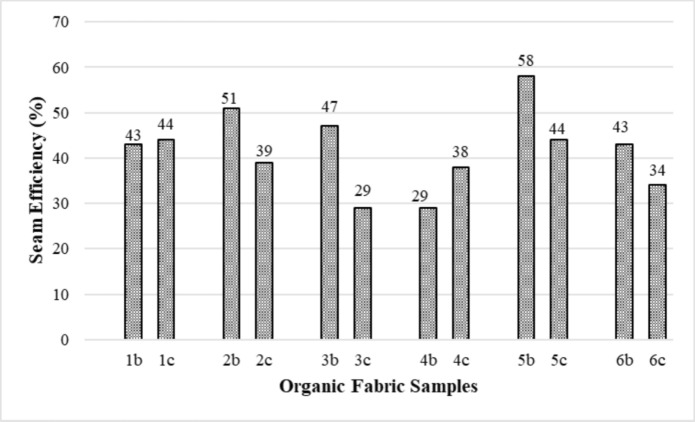
Seam efficiency results of the organic fabric samples

The seam efficiency of the fabrics is significant for the seam quality and seam regularity during clothing manufacturing. The higher the seam efficiency, the easier the sewing fabric becomes. According to the results obtained from Fig. 5, except for fabric group four, the seam efficiency of the organic fabric + natural dyed fabric samples in other samples was lower. This shows that the seam efficiency of the naturally dyed organic fabric samples was lower.

The Anova and SNK test results in Table 3 revealed that the fabric samples' weight and organic certificate/dye type are statistically significant in the seam strength and seam efficiency. Fabric groups with high weight (4th and 5th groups) have higher seam strength and seam efficiency than fabric groups with low weight. Fabric samples with organic certificate/reactive dye and organic certificate/natural dye have lower seam strength and seam efficiency than uncertificated/reactive dye fabric samples.

**Table 3:** Statistical analysis (ANOVA and SNK test) results for seam strength and seam efficiency

		Seam Strength		Seam Efficiency	
		P/Sig	SNK Ranges	P/Sig	SNK Ranges
Weight (g/m^2^)	1st group	.000*	394.81 d	.000*	54.82 b
	2nd group		358.33 c		57.51 b
	3rd group		257.25 a		40.61 a
	4th group		434.47 e		44.81 a
	5th group		441.82 e		55.74 b
	6th group		313.36 b		52.72 b
Organic certificate/ dye type	Uncertified-Reactive	.000*	513.47 c	.000*	69.18 c
	Organic GOTS/OCS-Reactive		316.84 b		45.26 b
	Organic GOTS/OCS- Natural		269.71 a		38.68 a

^*^: Statistically significant (P < 0.05)

(a), (b), (c),(d) and (e) represent statistically difference ranges according to the SNK test

### Biodegradability test results of fabric samples

**Fig. 6:**
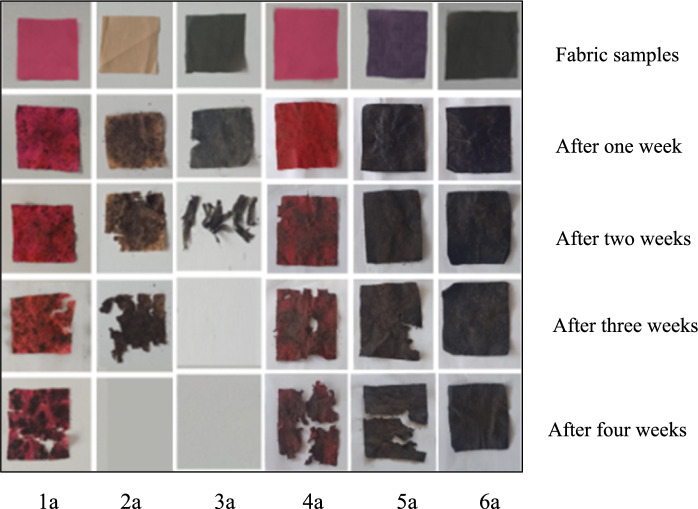
Biodegradability test results of conventional fabric + reactive dyed samples

**Fig. 7:**
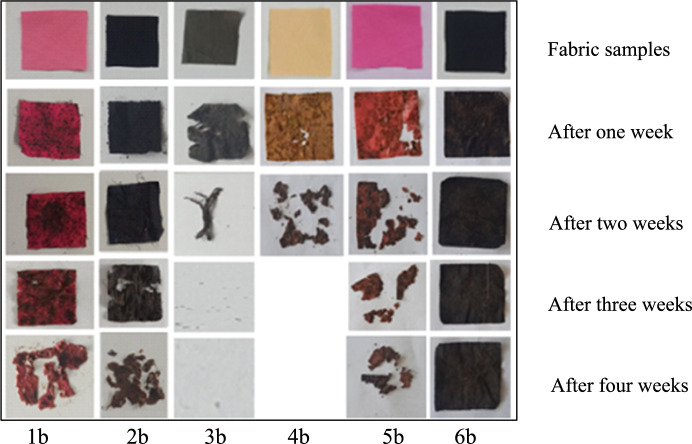
Biodegradability test results of organic fabric + reactive dyed samples

**Fig. 8:**
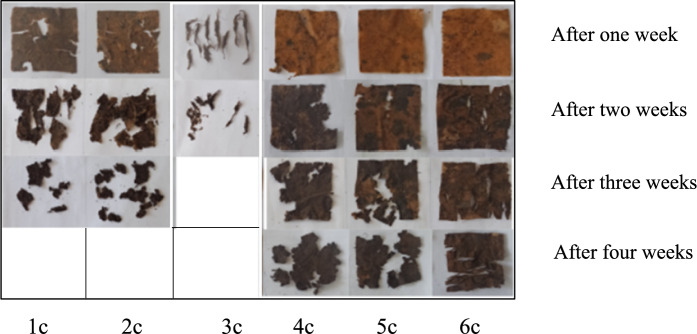
Biodegradability test results of organic fabric + nature dyed samples

The results of the soil burying test conducted to examine the biodegradability properties of the fabric samples were obtained by observation at 1-week intervals. Accordingly, it was observed that the dissolution time in the soil shortened as the weight of the fabric decreased. It was determined that the sample with viscose in its structure dissolved faster, and the sample with synthetic fibre in its structure decomposed later. It was observed that fabric samples, except for group 6 containing polyamide and elastane, degraded more quickly. The degradation stages of conventional fabric samples dyed with reactive dye in Fig. 6, organic fabric samples dyed with reactive dye in Fig. 7, and organic fabric samples dyed with natural dye in Fig. 8 are seen over four weeks. As seen in Fig. 8, naturally dye organic fabric samples decompose more quickly than other samples.

%100 pure organic cotton will biodegrade more quickly if the conditions are right; however, conventional cotton which has been heavily treated with chemicals may well take longer and as it does degrade will release chemicals into the ground.

### Images of women’s blouses sewn from fabric samples

**Fig. 9:**
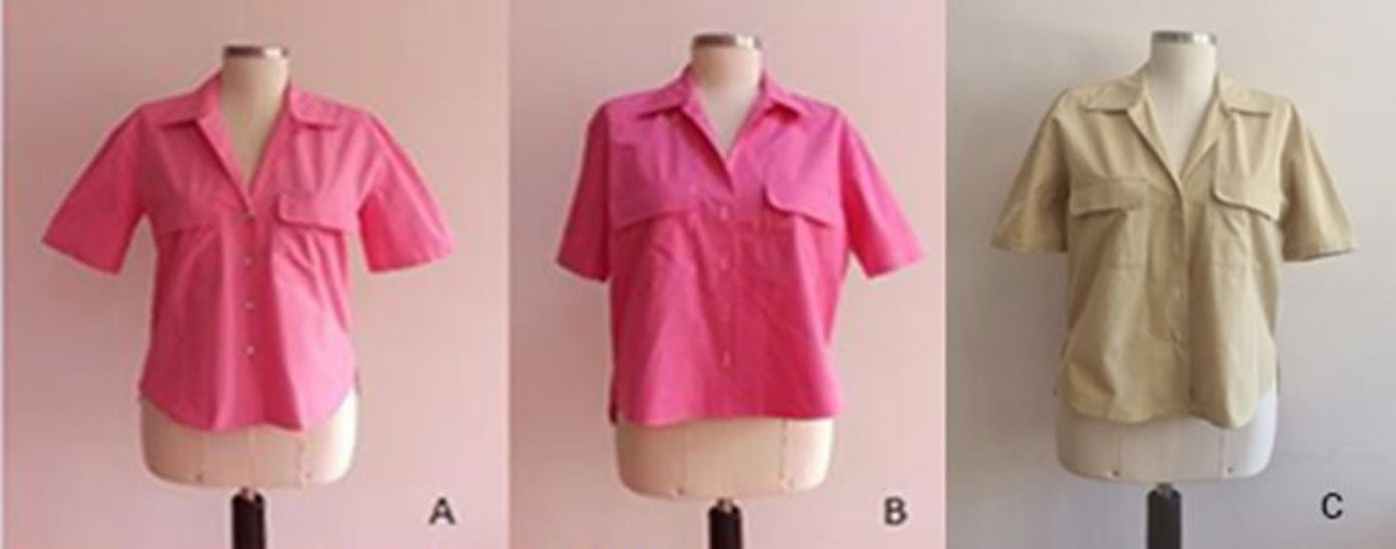
Women's blouse with fabric samples 1a, 1b and 1c

**Fig. 10:**
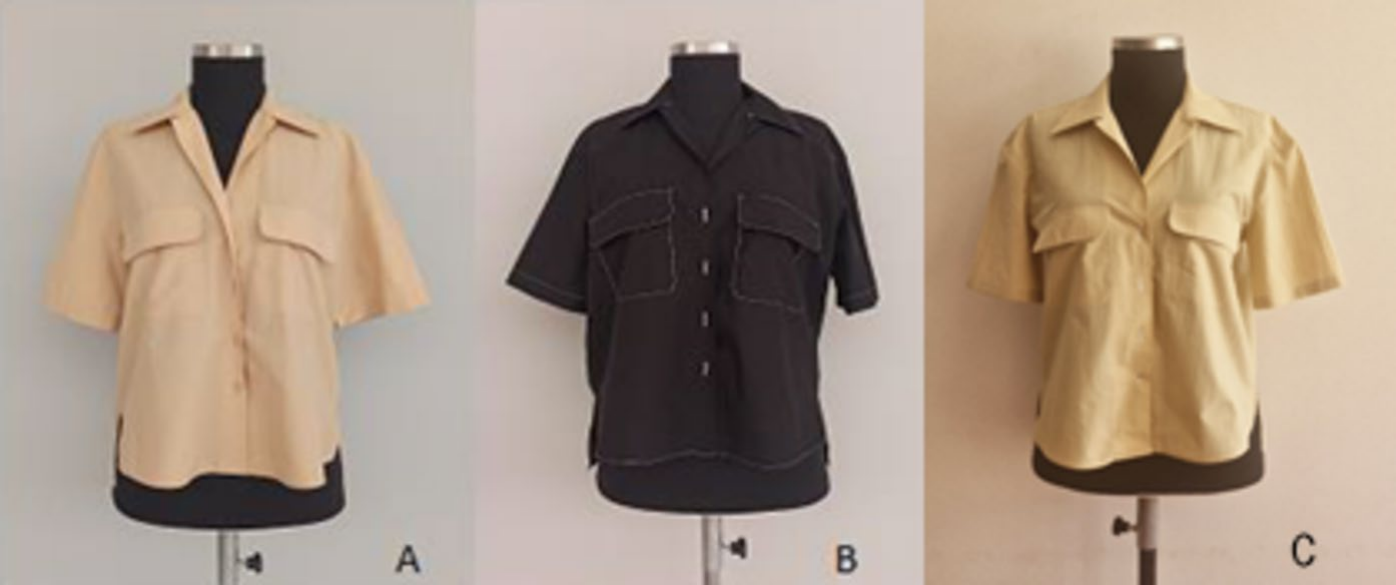
Women's blouse with fabric samples 2a, 2b and 2c

Photo images of blouses allow for assessing the ease, fit and appearance of the model. Photo images of blouses sewn from the fabrics in the first group are shown in Fig. 9 and blouses sewn from the fabrics in the second group are shown in Fig. 10. Subjective evaluation was used to evaluate the appearance of women's blouses from photographs. When the blouses sewn from a, b and c fabric samples in the first group in Fig. 9 are examined carefully, it is observed that the 1c blouse sample sewn from naturally dyed organic woven fabric looks more natural and rigid. In addition, it is observed that the 1a blouse sample sewn from reactive dyed conventional woven fabric looks more flowing.

When the blouses sewn from a, b and c fabric samples in the second group in Fig. 10 are examined carefully, it is observed that the 2c blouse sample sewn from naturally dyed organic woven fabric looks more natural, rigid and comfortable, too. Additionally, it was observed that blouse sample 2a, sewn from reactive dyed conventional woven fabric, appeared more flowing.

## Conclusion

In recent years, it has become important for a sustainable environment that clothes are biodegradable, and that unused clothing does not pollute the environment by decomposing in nature. Cotton, especially organic cotton, can decompose very quickly in nature. Since organic clothing production has become important in recent years, the seam performance and biodegradability of these products, unlike conventional products, are important in creating new data for the textile and clothing industry*.* This study aims to examine seam performance and biodegradability of clothes produced from organic cotton woven fabric that are not harmful to the environment and humans.

As seen from the results obtained from this study, fabric weight and conventional or organic cotton with reactive dye and natural dye were significant factors in fabric breaking strength, seam strength, and seam efficiency properties of cotton woven samples at a significant level of 0.05. Anova and SNK test results indicated that conventional cotton woven fabric samples revealed higher breaking strength, higher seam strength, and seam efficiency values compared to organic cotton woven fabric samples. Anova and SNK test results also indicated that organic cotton fabric with natural dye samples revealed lower breaking strength, seam strength and seam efficiency values than organic cotton with reactive dye samples. Since polyester sewing thread is used in blouse samples sewn from reactive-dyed conventional fabrics, their seam strength and seam efficiency are higher than blouse samples sewn from organic fabric with cotton sewing thread.

When the biodegradability of the fabric samples used in the experimental study is examined, it is seen that the naturally dyed organic fabric samples decompose the fastest.

When the blouses sewn from a, b and c fabric samples in the first group in Fig. 10 are examined carefully, it is observed that the 1c blouse sample sewn from naturally dyed organic woven fabric looks more natural, rigid and comfortable. The blouses sewn from reactive-dyed conventional woven fabrics look more flowing.

Organic cotton woven fabric is an excellent choice for eco-conscious consumers and fashion enthusiasts alike, offering a unique blend of sustainability, comfort, and style. Finally, it can be concluded that from the sustainability, quality and biodegradability, organic fabric with natural dyed samples for clothes is easily acceptable as an alternative to conventional fabric with reactive dyed samples.

## Data Availability

No datasets were generated or analysed during the current study.
